# *Q*-score as a reliability measure for protein, nucleic acid and small-molecule atomic coordinate models derived from 3DEM maps

**DOI:** 10.1107/S2059798325005923

**Published:** 2025-07-14

**Authors:** Grigore Pintilie, Chenghua Shao, Zhe Wang, Brian P. Hudson, Justin W. Flatt, Michael F. Schmid, Kyle L. Morris, Stephen K. Burley, Wah Chiu

**Affiliations:** a Departments of Bioengineering and of Microbiology and Immunology Stanford University Stanford CA94305 USA; b Research Collaboratory for Structural Bioinformatics Protein Data Bank, Institute for Quantitative Biomedicine Rutgers, The State University of New Jersey Piscataway NJ08854 USA; c European Molecular Biology Laboratory European Bioinformatics Institute (EMBL–EBI) Wellcome Genome Campus HinxtonCB10 1SD United Kingdom; d Rutgers Artificial Intelligence and Data Science (RAD) Collaboratory Rutgers, The State University of New Jersey Piscataway NJ08854 USA; e Rutgers Cancer Institute, New Brunswick, NJ08903, USA; f Research Collaboratory for Structural Bioinformatics Protein Data Bank, San Diego Supercomputer Center University of California, San Diego La Jolla CA92093 USA; g Department of Chemistry and Chemical Biology Rutgers, The State University of New Jersey Piscataway NJ08854 USA; h Division of Cryo-EM and Bioimaging, SSRL SLAC National Accelerator Laboratory Menlo Park CA94025 USA; Rutherford Appleton Laboratory, United Kingdom

**Keywords:** cryoEM, *Q*-scores, validation, *B* factors, structure

## Abstract

Atomic coordinate models are important for the interpretation of 3D maps produced with cryoEM and cryoET (3D electron microscopy; 3DEM). In addition to visual inspection of such maps and models, quantitative metrics can inform about the reliability of the atomic coordinates, in particular how well the model is supported by the experimentally determined 3DEM map. A recently introduced metric, *Q*-score, was shown to correlate well with the reported resolution of the map for well fitted models. Here, we present new statistical analyses of *Q*-score based on its application to ∼10 000 maps and models archived in the EMDB (Electron Microscopy Data Bank) and PDB (Protein Data Bank). Further, we introduce two new metrics based on *Q*-score to represent each map and model relative to all entries in the EMDB and those with similar resolution. We explore through illustrative examples of proteins, nucleic acids and small molecules how *Q*-scores can indicate whether the atomic coordinates are well fitted to 3DEM maps and also whether some parts of a map may be poorly resolved due to factors such as molecular flexibility, radiation damage and/or conformational heterogeneity. These examples and statistical analyses provide a basis for how *Q*-scores can be interpreted effectively in order to evaluate 3DEM maps and atomic coordinate models prior to publication and archiving.

## Abstract 2

*Q*-scores are calculated for atomic models derived from 3D electron microscopy maps, measure how well the model fits the map and reflect the quality of the map itself. Here, we develop a statistical model for *Q*-scores applied to many maps and models in the EMDB and PDB, respectively, and show how it can be used to assess the reliability of entire models as well as their subcomponents.

## 1. Introduction

Atomic coordinate models derived from 3DEM maps give many insights into the structure and function of biological macromolecules. Building models into 3DEM maps can take various paths, such as the fitting of known models obtained previously with experimental methods (Pintilie & Chiu, 2012 ▸) or predicted with computational methods such as *AlphaFold* (Jumper *et al.*, 2021 ▸). Alternatively, in near-atomic resolution maps, models can be built *de novo* either interactively (Casañal *et al.*, 2020 ▸) or automatically (Jamali *et al.*, 2024 ▸). The quality of the 3DEM map can vary locally (Vilas *et al.*, 2020 ▸), and it has become more critical to quantitatively assess the reliability of models and their various molecular components, which can be accomplished by the application of map–model metrics.

An example of a map–model metric is atom inclusion, an early metric which is still used in validation reports for depositions to the Electron Microscopy Data Bank (EMDB; Joseph *et al.*, 2017 ▸; wwPDB Consortium, 2024 ▸). Other map–model metrics include cross-correlation (Klaholz, 2019 ▸), mutual information (Vasishtan & Topf, 2011 ▸), *EM-Ringer* (Barad *et al.*, 2015 ▸) and FSC-Q (Ramírez-Aportela *et al.*, 2021 ▸). A recent Community Challenge in which many worldwide groups participated has compared such metrics, showing some similarities and correlations amongst them (Lawson *et al.*, 2021 ▸). For example, the *Q*-score metric was shown to correlate well with the reported map resolution and hence relates to resolvability. However, low *Q*-scores may also be observed when the model is not fitted properly to the map, or when a group of atoms may not be resolved due to flexibility, radiation damage or different charged states of a group of atoms (Burley *et al.*, 2022 ▸; Pintilie *et al.*, 2020 ▸). Regardless of the reason, the *Q*-score applies individually to each atom, indicating the degree to which the atom is resolved in the map based on the map values around it.

While *Q*-scores have already been added to validation reports for maps and models deposited in the EMDB (Kleywegt *et al.*, 2024 ▸), here we continue to evaluate how they may be interpreted in several contexts. For example, *Q*-scores can be averaged over all atoms in an entire model, in individual protein residues (Pintilie & Chiu, 2021 ▸) and nucleotides (Kretsch *et al.*, 2025 ▸), or in small molecules such as ligands (Lawson *et al.*, 2024 ▸), saccharides (Chmielewski *et al.*, 2023 ▸) and lipids (Chmielewski *et al.*, 2024 ▸). We show how the averaged *Q*-scores can be interpreted based on statistics derived from ∼10 000 map/model combinations freely available from the EMDB and PDB.

In particular, we carry out a comprehensive statistical analysis of how *Q*-scores are related to reported resolution, based on ∼10 000 EMDB maps and associated PDB atomic coordinate models archived in the EMDB. The purpose of this study is to establish statistically sound metrics useful for evaluating 3DEM maps and models of biomolecules, including proteins, nucleic acids and small-molecule ligands. As *Q*-scores are already included in wwPDB validation reports, another goal is to provide new percentile-based formulations to be used in such a context. The percentile *Q*-score-based metrics introduced here are meant to indicate how a map and model combination compares with other 3DEM maps and models in the EMDB and PDB, and thus serve as an indication of map and model quality relative to all of the publically available 3DEM structures (Gore *et al.*, 2017 ▸; Feng *et al.*, 2021 ▸).

We also further explore the use of *Q*-scores to derive atomic *B* factors. Atomic *B* factors have been commonly used in macromolecular crystallography (MX), and are also known as Debye–Waller factors (Winn *et al.*, 2001 ▸) or atomic displacement parameters (Afonine *et al.*, 2018 ▸). In 3DEM, the term *B* factor is also used to describe the overall decay of high-frequency information due to electron-microscope parameters and detector-performance factors (Rosenthal & Henderson, 2003 ▸), and also to report the amount of sharpening applied to a map to improve visualization in real space (Kaur *et al.*, 2021 ▸). Here, we use the term atomic *B* factor to distinguish their application to individual atoms in models fitted to 3DEM maps. In the field of 3DEM, atomic *B* factors can be calculated during model refinement (Afonine *et al.*, 2018 ▸; Beton *et al.*, 2024 ▸) or molecular-dynamics flexible fitting (Frank, 2017 ▸). We showed previously that atomic *B* factors can also be derived from *Q*-scores (Zhang, Pintilie *et al.*, 2020 ▸; Pintilie & Chiu, 2021 ▸). Here, we expand this analysis with more examples, showing that atomic *B* factors can confidently be derived from *Q*-scores at resolutions ranging from ∼1 to ∼4 Å.

## 2. *Q*-scores of maps and models in the EMDB and PDB

**Figure 1:**
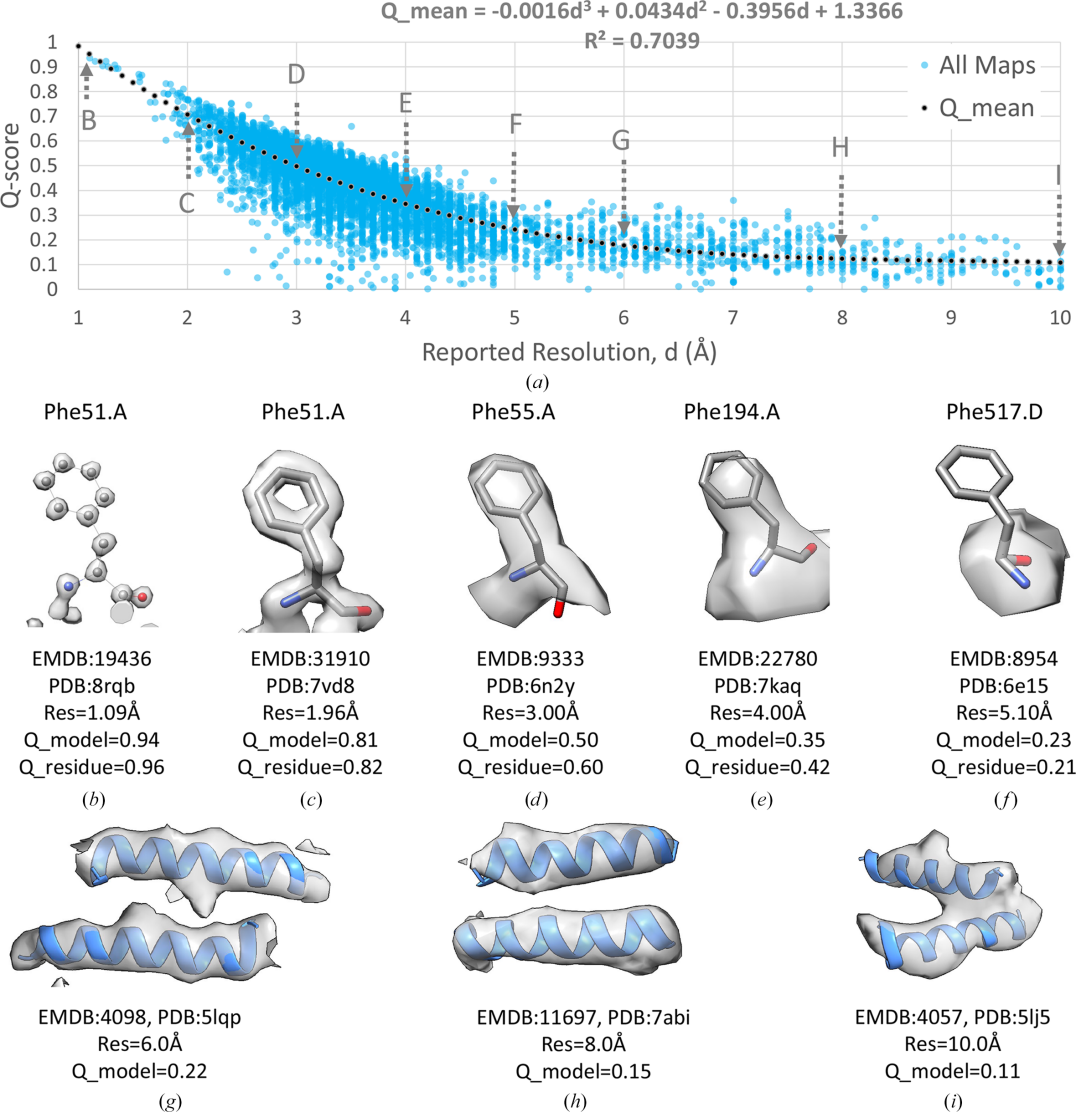
Relationship between *Q*-score and reported resolution, *d*, using EMDB maps and their associated atomic models in the PDB. (*a*) A plot showing each map and model pair as a filled circle, with a dotted line showing a regression using a third-degree polynomial. (*b*–*f*) Side chains at various resolutions, with corresponding decreasing *Q*-scores, averaged over the whole model (*Q*_model) or averaged over the residue shown (*Q*_residue). (*g*–*i*) α-Helices at three different resolutions between 5 and 10 Å.

*Q*-scores were calculated for 10 189 map/model combinations in the EMDB and PDB, selecting primarily maps with reported resolution between 1 and 10 Å using the gold-standard FSC_0.143_ criterion (Henderson *et al.*, 2012 ▸). The *Q*-score averaged over all non-H atoms in a model is plotted against the reported resolution in Fig. 1 ▸(*a*). A regression of these data points using a third-degree polynomial (Fig. 1 ▸) shows good correlation, with *R*^2^ = 0.7039. Residual plots in Supplementary Fig. S1 confirm that this relationship fits the data well. We used a third-degree polynomial because it fits the data better with higher *R*^2^ than do linear (*R*^2^ = 0.5959) or second-degree polynomial (*R*^2^ = 0.6999) regressions, while not overfitting the data. Using a fourth-degree polynomial did not significantly improve the fit (*R*^2^ = 0.7061). The third-degree polynomial model was also verified as the optimal polynomial regression calculation by cross-validation and visual inspection of regression residual plots (Appendix *A*[App appa], Sections A1.2 and A1.3).

The plot in Fig. 1 ▸ shows that *Q*-scores decrease quickly from ∼1 to ∼0.3 for maps with resolutions of 1–5 Å and they decrease more slowly from ∼0.3 to ∼0.1 for maps with resolutions of 5–10 Å. Figs. 1 ▸(*b*)–1 ▸(*i*) show examples of maps and models with average *Q*-scores near the regression line, illustrating that *Q*-scores correlate well with the resolvability of atoms and groups of atoms such as protein residues and α-helices. For example, *Q*-scores near ∼1.0 are associated with individually resolved atoms (Fig. 1 ▸*b*) and *Q*-scores near ∼0.5 are associated with resolved side chains in protein residues (Fig. 1 ▸*d*). *Q*-scores near ∼0.2 are associated with unresolved side chains but resolved secondary structures such as α-helices in proteins (Fig. 1 ▸*f*–1 ▸*i*).

Fig. 1 ▸ shows some data points far away from the regression line, especially those far below the line, with *Q*-scores close to 0, for example in the resolution range 2.5–5 Å. In Supporting Information S1 we detail how removing some of these outliers using cross-correlation scores yields similar regression curves.

## 3. Statistical model for *Q*-scores

In Supporting Information S2 we detail how we arrive at the following equations for characterizing *Q*-scores at different resolutions using the polynomial regression curve illustrated in Fig. 1 ▸:
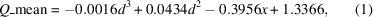

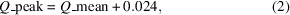

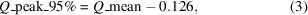

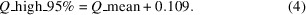


In equation (1), *Q*_mean represents the mean *Q*-score value as a function of reported resolution, *d*, as calculated by regression with the third-degree polynomial curve illustrated in Fig. 1 ▸. In equations (2), (3) and (4), offsets act to move the *Q*_mean curve up and down to three specific positions. The first is *Q*_peak (equation 2), which positions the curve such that the highest number of data points are close to the line (within a window size of 0.01). The other two positions are *Q*_low_95% (equation 3) and *Q*_high_95% (equation 4). These two latter offsets move the curve to positions such that 95% of the data points fall between them and *Q*_peak.

*Q*_peak represents the *Q*-score observed in the highest number of map–model pairs, based on the set of ∼10 000 maps in the EMDB considered here. In statistics, this is also often called the mode of the distribution. For a normal distribution, the mean is considered to be the expected value and coincides with the peak of the curve. However, in this case, because the distribution is skewed (as shown in Supplementary Fig. S3*b*), the mean does not coincide with the peak. The other two curves, *Q*_low_95% and *Q*_high_95%, provide two *Q*-scores below/above which a small fraction of maps (5%) are observed. Below and above these curves, *Q*-scores may be ‘outliers’ or ‘not commonly observed’ for a given reported resolution.

**Figure 2:**
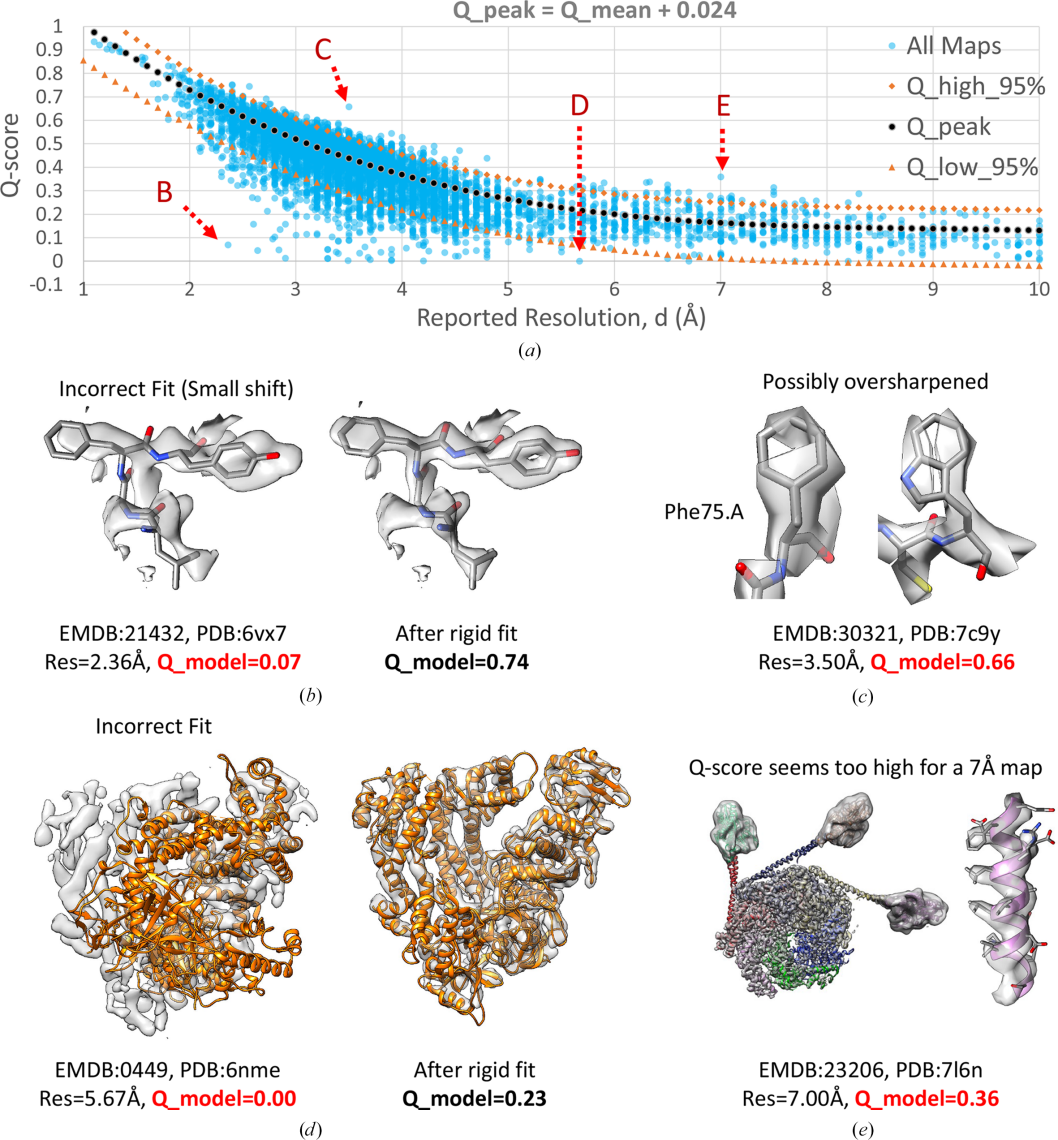
(*a*) Plot of *Q*-scores versus reported resolution for ∼10 000 maps and models in the EMDB (the same data set as in Fig. 1 ▸). The dotted curves above and below the *Q*_peak curve enclose 95% of the data points (equations 2, 3 and 4). (*b*–*e*) Illustration of maps and models with *Q*-scores outside the 95% curves. Overall *Q*-scores for each model are indicated with *Q*_model and are colored red if outside the 95% curves. In (*b*) and (*d*) the *Q*-scores are inside the 95% curves after properly fitting the model to the map and re-calculating the *Q*-scores.

Fig. 2 ▸(*a*) shows the same plot as in Fig. 1 ▸(*a*), with all ∼10 000 map–model pairs, also plotting the *Q*_peak, *Q*_high_95% and *Q*_low_95% curves. Several outliers which are outside the 95% curves are shown in Figs. 2 ▸(*b*)–2 ▸(*e*). In Figs. 2 ▸(*b*) and 2 ▸(*d*), maps and models with *Q*-scores lower than *Q*_low_95% are shown. These appear to have low *Q*-scores due to the model not being fitted correctly to the map. Correct fitting brings the *Q*-scores within the 95% range.

Fig. 2 ▸(*c*) shows an example where the *Q*-score is above the *Q*_high_95% line and hence may also be considered to be an outlier. The map appears discontinuous and noisy, indicating that the map is likely oversharpened. While severe oversharpening was shown to yield lower *Q*-scores due to excessive noise, a small amount of oversharpening may raise *Q*-scores, especially if the model is refined into the oversharpened map. Fig. 2 ▸(*e*) shows another outlier where the *Q*-score is above the *Q*_high_95% curve. In this case, most of the map appears to be resolved at higher resolution. Hence, in this case the reported resolution is likely to be underestimated and does not reflect the overall resolvability of all the features in the map.

In Supporting Information S3, we also show how we can use a rolling-window approach over the same data set to derive similar percentile statistics without using the polynomial regression curve. The two approaches are shown to produce very similar results; however, using the polynomial regression curve method appears to produce smoother curves for *Q*_high_95% and *Q*_low_95%, which is advantageous.

## 4. Per-residue and per-nucleotide *Q*-scores

**Figure 3:**
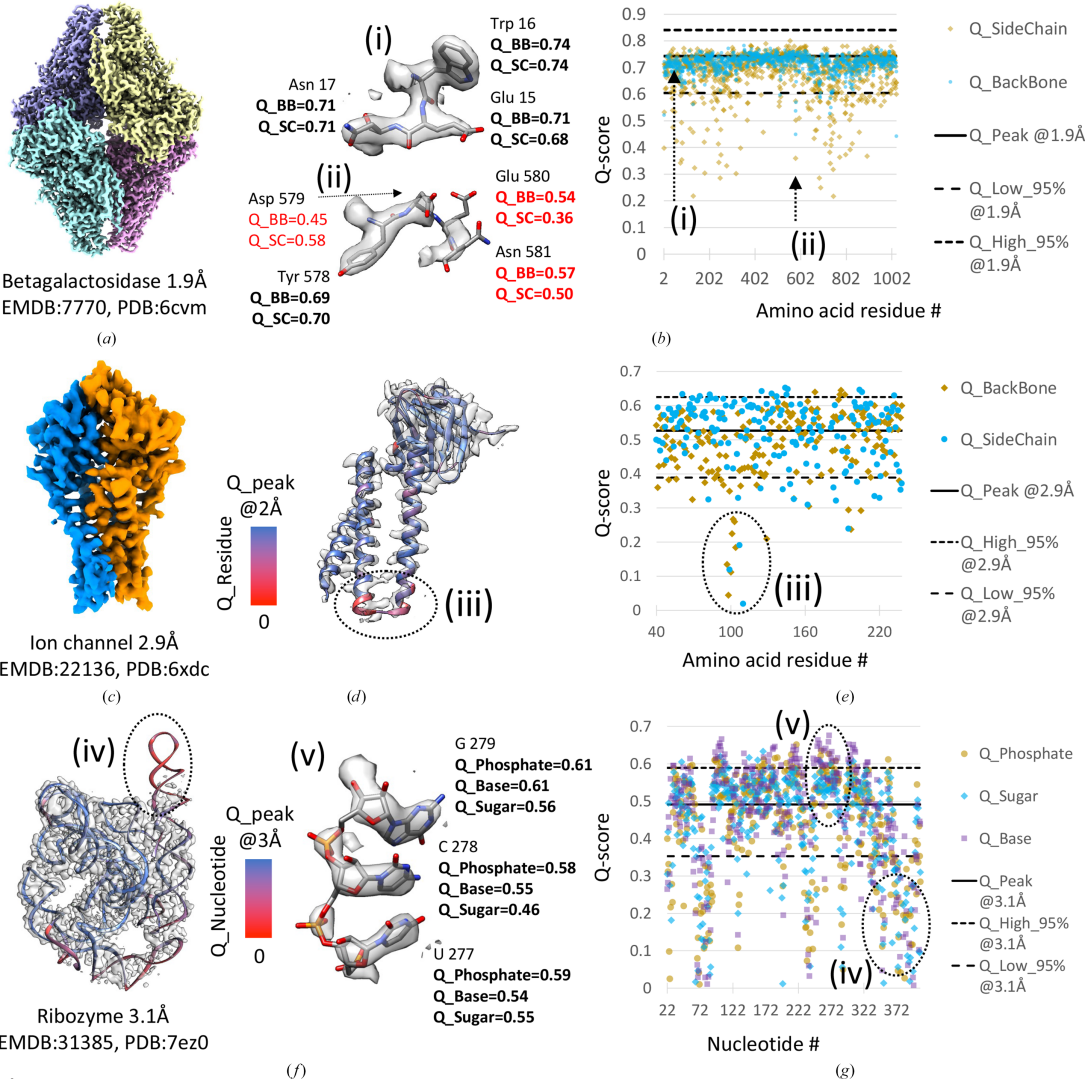
Examples of *Q*-score application in proteins and in nucleic acids. (*a*) β-Galactosidase protein complex with (*b*) per-residue backbone and side-chain *Q*-scores; example residues with *Q*-scores marked on the plot are marked (i) and (ii). (*c*) Ion-channel protein complex; one of the two proteins in the complex is shown in (*d*), with a ribbon display color-coded by residue *Q*-score. (*e*) Per-residue backbone and side-chain *Q*-scores for one ion-channel complex protein; an area with low *Q*-scores is marked (iii). (*f*) RNA-only *Tetrahymena* ribozyme; the ribbon model is color-coded by nucleotide *Q*-score. (*g*) *Q*-scores of phosphate, sugar and base atoms in each nucleotide; *Q*-scores for three residues which are well resolved are shown in (v) and an area with low nucleotide *Q*-scores is marked (iv).

*Q*-scores are calculated for each atom, but they can also be averaged over all atoms in a model (as in the previous analyses) and also for groups of atoms within protein amino-acid residues or nucleic acid nucleotides. We illustrate this in the examples below. Fig. 3 ▸(*a*) shows a segmented map of β-galactosidase imaged at 1.9 Å resolution (EMDB entry EMD-7770; Bartesaghi *et al.*, 2018 ▸). In Fig. 3 ▸(*b*), *Q*-scores of backbone and side-chain atoms are plotted for every residue in the associated model with PDB entry 6cvm. *Q*-scores of backbone atoms are mostly close to the *Q*_peak line calculated with equation (2). Side-chain atoms, however, have more variable *Q*-scores, some of which are below the *Q*_low_95% line calculated with equation (3). Residues with low *Q*-scores for backbone and/or side-chain atoms can be identified in such a plot, as in example (ii) shown in Figs. 3 ▸(*a*) and 3 ▸(*b*), where low *Q*-scores are labeled in red. This can be used to identify areas of the map where the model may not be fitted properly, or where the map is not well resolved and hence the accuracy of these parts of the model may be low.

Fig. 3 ▸(*c*) illustrates a 2.9 Å resolution map of a SARS-CoV-2 ion channel (EMDB entry EMD-22136; Kern *et al.*, 2021 ▸). In Fig. 3 ▸(*d*) per-residue *Q*-scores are used to color-code the backbone ribbon of one of the proteins, with red corresponding to low *Q*-scores (near 0) and blue corresponding to *Q*-scores near *Q*_peak (as commonly observed for this resolution). *Q*-scores of backbone and side-chain atoms in each residue are also plotted in Fig. 3 ▸(*e*); most fall within the 95% bounds. An area where *Q*-scores are much lower is marked (iii) in Fig. 3 ▸(*e*); it can also be seen as a red-colored ribbon in Fig. 3 ▸(*d*), corresponding to low *Q*-scores. This display can be very useful for identifying areas where the map is not well resolved due to conformational heterogeneity or where the atomic coordinate model may need further refinement to better fit the map.

Fig. 3 ▸(*f*) shows a 3DEM map of the RNA-only *Tetrahymena* ribozyme reconstructed to 3.1 Å resolution (EMDB entry EMD-31385; Su *et al.*, 2021 ▸). Per-nucleotide *Q*-scores are plotted in Fig. 3 ▸(*g*). *Q*-scores were averaged and plotted for base, ribose and phosphate atoms in each nucleotide. An area where *Q*-scores are much lower than commonly observed, under the *Q*_low_95% line, is marked (iv); the corresponding area in the map is not resolved well, likely due to conformational heterogeneity. An area where nucleotides are resolved as expected, and correspondingly where *Q*-scores are above the *Q*_peak line, is marked (v).

## 5. *Q*-scores for small molecules

**Figure 4:**
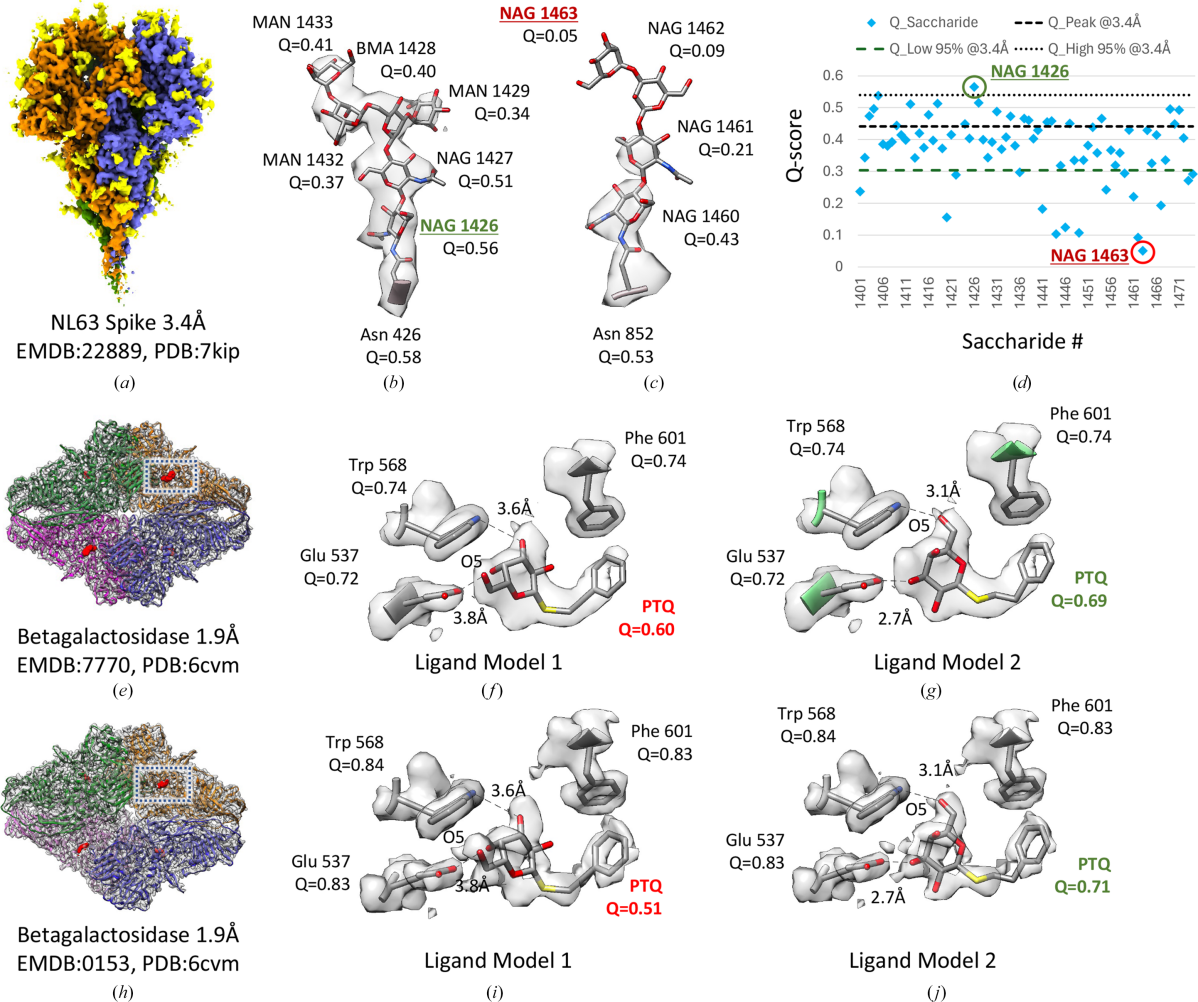
Application of *Q*-scores to small molecules. (*a*) Segmented 3DEM map of coronavirus NL63 spike proteins (blue, orange, green) with Asn-associated glycans (yellow). (*b*, *c*) Two example glycans, with *Q*-scores for each component saccharide. (*d*) The *Q*-scores of each saccharide are plotted. (*e*, *h*) Two 3DEM maps of β-galactosidase with the same reported resolution of 1.9 Å. Two models of the ligand PTQ and three interacting protein residues, along with *Q*-scores, are shown in (*f*) and (*g*) for the map in (*e*) and in (*i*) and (*j*) for the map in (*h*).

*Q*-scores can also be calculated for small molecules to inform whether their atomic coordinates are well resolved and/or fitted correctly in the 3DEM map. An example is a glycan made up of smaller oligosaccharide molecules covalently bonded to proteins such as the NL63 spike trimer (Zhang, Li *et al.*, 2020 ▸). In Fig. 4 ▸(*a*), a segmented 3DEM map of the coronavirus NL63 (EMDB entry EMD-22889) shows the three spike proteins with Asn-associated glycans in yellow. Fig. 4 ▸(*d*) plots *Q*-scores of each saccharide molecule. Most of the saccharide units are resolved, with *Q*-scores within the 95% bounds, as in the example in Fig. 4 ▸(*b*). At the same time, from the *Q*-score plot it is easy to identify those that are not well resolved, as shown in Fig. 4 ▸(*c*), likely due to conformational heterogeneity.

As another example, we computed *Q*-scores for the PTQ ligand in the β-galactosidase complex (Bartesaghi *et al.*, 2018 ▸). Figs. 4 ▸(*e*) and 4 ▸(*h*) show two maps of this complex with the same reported resolution of 1.9 Å. In a recent 3DEM ligand-modeling challenge (Lawson *et al.*, 2024 ▸), participants reported two potential models for this ligand in the target 3DEM map EMDB entry EMD-7770. The two models are shown in Figs. 4 ▸(*f*) and 4 ▸(*g*). The O5 atom in the ligand is marked in both images to show the difference, which is that the pyranose ring is flipped ∼180° in one model relative to the other. We also fitted these two models to the map of the same complex, EMDB entry EMD-0153, shown in Fig. 4 ▸(*h*); the fitted ligands are shown in Figs. 4 ▸(*i*) and 4 ▸(*j*). We calculated *Q*-scores for both ligand models in both maps. Model 1 has lower *Q*-scores in both maps, near or under the *Q*_low_95% value, and hence may be considered an outlier or unlikely. On the other hand, model 2 has higher *Q*-scores in both maps, in line with *Q*-peak or the commonly observed *Q*-score at this resolution; it also shows more favorable interaction distances with two nearby residues, as shown in Figs. 4 ▸(*g*) and 4 ▸(*j*). Taken together, this indicates that model 2 is more likely to be correct.

## 6. *Q*-scores versus *B* factors

When generating a 3D map from atomic coordinates (a model map), the effect of atomic *B* factors is to spread out the map values around the position of each atom. The higher the *B* factor of an atom, the more diffuse or blurry, and the less sharp, the surrounding map values around the atom are. This effect can be characterized by *Q*-scores, because *Q*-scores are higher for sharper peaks and lower for more diffuse peaks. Hence, we use a scaling parameter to calculate atomic *B* factors from *Q*-scores, using the equation
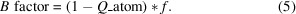


In equation (5), the scaling factor *f* is determined by maximizing the similarity between the 3DEM map and the model map generated using the resulting *B* factors. Model maps are generated with atomic *B* factors resulting from scaling factors in the range 0–300, and compared with the 3DEM map by cross-correlation around the mean (CC-mean). The optimal scaling factor *f* and resulting atomic *B* factors are those that yield the highest cross-correlation score between the model map and the 3DEM map.

**Figure 5:**
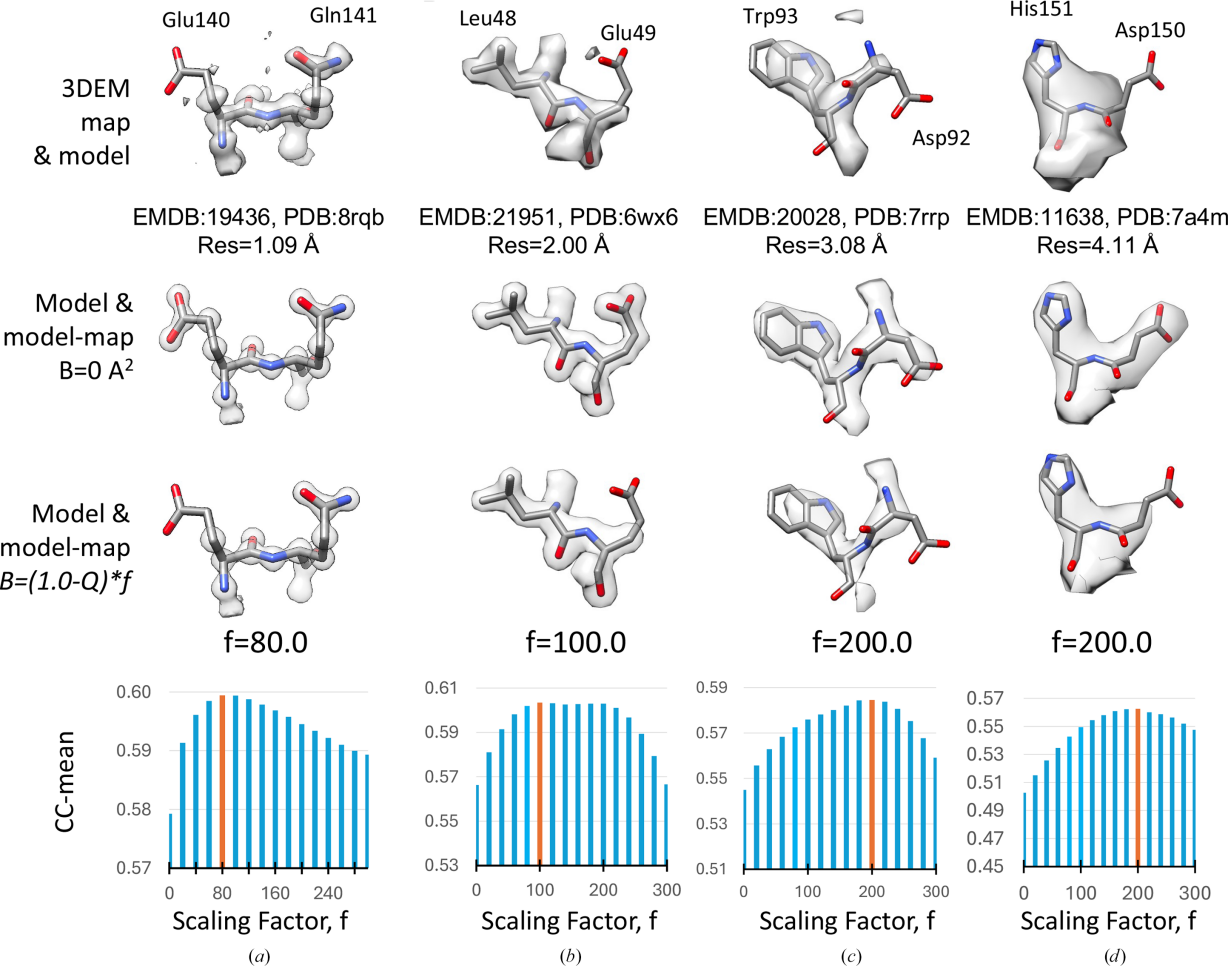
Atomic *B* factors from *Q*-scores. Top row: two residues in four different 3DEM maps and models with resolutions of ∼1 to ∼4 Å. Second row: model maps generated with atomic *B* factors calculated by scaling *Q*-scores. Third row: model maps generated with atomic *B* factors set to 0. Fourth row: bar plots of CC-mean (cross-correlation about the mean) between the 3DEM map and model maps generated with atomic *B* factors calculated using a range of scaling factors (0–300); the bar with the highest CC-mean value is colored orange.

Fig. 5 ▸ (top row) shows residues from four different 3DEM maps and models with resolutions in the range of ∼1 to ∼4 Å. When using *B* factors of 0 Å^2^, all residues and side chains are resolved equally (Fig. 5 ▸, second row), but this does not look like the 3DEM map, where some residues are not resolved. When using *B* factors derived from *Q*-scores using the optimal scaling factor, the model map looks more like the 3DEM map (Fig. 5 ▸, third row): side chains that are not resolved in the 3DEM map (and hence have low* Q*-scores, which would result in a high *B* factors) are also not resolved in the model map. Fig. 5 ▸ (bottom row) shows plots of the CC-mean obtained with different scaling factors for each of the four examples, from which the optimal scaling factor (colored in orange and shown above the plot) is determined.

## 7. Relative *Q*-scores

Relative *Q*-scores aim to compare a map–model entry with other entries in the EMDB. Here, we introduce two new terms: *Q*_relative_all and *Q*_relative_resolution. *Q*_relative_all expresses the *Q*-score of a map–model entry as a percentile relative to all of the entries in the EMDB, while *Q*_relative_resolution expresses it relative to entries with similar resolutions.

*Q*_relative_all is defined for a map–model pair with *Q*-score *Q* as follows:
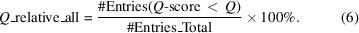


In equation (6), the numerator represents the number of EMDB entries with *Q*-scores lower than that of the entry in question, and the denominator is the total number of entries in the EMDB. *Q*_relative_all thus represents the percentile ranking of an entry within the entire data set of EMDB entries.

*Q*_relative_resolution is defined for a map–model pair with *Q*-score *Q* and resolution *d* as follows:
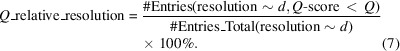


In equation (7), the numerator represents the number of EMDB entries which have resolution close to *d*, more specifically within a window size *w* of the reported resolution of the entry, and also which have a lower *Q*-score than the *Q*-score of the entry, *Q*. The denominator is the total number of entries which have resolution within the same window size *w* of the resolution of the entry, *d*.

We address here what would be a good resolution-window size (*w*) for comparing entries for calculating *Q*_relative_resolution. To test the effect of this resolution window size, we selected 12 window sizes ranging from 0.1 to 1.0 Å, with increments of 0.1 Å, including additional sizes of 1.2 and 1.5 Å. As shown in Table 1 ▸, the number of entries (minimum, mean and maximum) increases with increasing window size. A larger number of entries for a given resolution would be more desirable for more meaningful statistical comparison.

A low correlation between *Q*_relative_resolution and reported map resolution would also be desirable, so that within each window *Q*_relative_resolution is not biased towards higher reported resolution entries. Thus, we tested the correlation between *Q*_relative_resolution and reported resolution for different window sizes. The Pearson correlation coefficient between resolution and *Q*_relative_resolution is plotted in Supplementary Fig. S5. Two curves are plotted: one for the correlation between *Q*_relative_resolution and reported map resolution, considering entries with resolutions higher than 5 Å (blue curve), and one considering entries with resolutions lower than 5 Å (red curve). For entries at resolutions lower than 5 Å, there is no significant correlation between *Q*_relative_resolution and reported resolution for all window sizes, as the correlation coefficient stays below 0.2 for all window sizes. However, for maps with resolutions higher than 5 Å (inclusive), negative correlations of higher magnitude are observed as the window size increases. Notably, at a window size of 0.5 Å the correlation nears −0.3, which represents a weak degree of correlation (Evans, 1996 ▸). Thus, for little or no correlation between *Q*_relative_resolution and reported resolution, according to Supplementary Fig. S5, the window size should be 0.5 Å or lower.

Some example values of *Q*_relative_all and *Q*_relative_resolution for the maps and models presented in Figs. 2 ▸ and 3 ▸ are shown in Table 2 ▸. For *Q*_relative_all, the higher the number, the higher the *Q*-score, and thus the better the overall quality of the map and model. On the other hand, *Q*_relative_resolution shows how the *Q*-score compares with other maps and models at similar resolution. The closer it is to 50%, the more it is ‘as commonly observed’. This would indicate a proper fit of the model to the map, and also an appropriate reported resolution value for the map. When *Q*_relative_resolution is much lower than this, for example lower than 5%, it could potentially indicate an incorrect fit of the model to the map, or a map at lower resolution than reported (as shown in Figs. 2 ▸*b* and 2 ▸*d*). When *Q*_relative_resolution it is much higher (for example 95% or more) it could potentially indicate other issues such as oversharpening of the map (as shown in Fig. 2 ▸*c*), or potentially that the reported resolution could be too low and does not reflect the overall map quality (as shown in Fig. 2 ▸*e*).

## 8. Summary and discussion

We previously showed that *Q*-scores correlate with reported resolutions of 3DEM maps for a small but representative number of maps and models (Pintilie *et al.*, 2020 ▸; Burley *et al.*, 2022 ▸). Here, we further expanded the data set to ∼10 000 maps and models at resolutions between 1 and 10 Å in the EMDB/PDB. We found that *Q*-scores correlate similarly to the reported resolution for this larger data set. Moreover, the distribution is close to normal but slightly skewed towards lower *Q*-scores, likely due to some models not being optimally fitted to the corresponding maps, and also potentially because maps may have regions with resolutions lower than the reported resolution. We derived a statistical model which provides, for a given resolution, the most commonly observed value, *Q*_peak, and also the 95% bounds *Q*_low_95% and *Q*_high_95%. The latter can be used to evaluate whether a calculated *Q*-score is as commonly observed/expected for a given resolution of the map or instead is more of an outlier if it is outside the 95% bounds.

We also note that the number of maps in the EMDB at each resolution varies (Supplementary Fig. S6), and this could affect the regression and statistical model. However, regression with a smaller data set including a similar number of maps and models at each resolution did not differ significantly from the regression with all 10 000 maps and models (Supplementary Fig. S2), indicating a robust regression model. Further, cross-validation results also indicate a reliable model (Section A1.3). We aim to update the regression and percentile bounds as more maps and models are deposited in the EMDB.

We showed how this statistical model can be used to contextualize *Q*-scores for entire models and also for smaller groups of atoms. *Q*-scores of groups of atoms can indicate whether protein residues, nucleotides or small ligands are resolved as expected given the reported resolution of the map. *Q*-scores can be low if the atom or groups of atoms are not resolved well, but also if they are not optimally fitted to the map, if they are flexible, if they are radiation damaged or if they have charged atom groups, as these factors affect the observed map values. *Q*-scores may also be affected by atom type (for example N versus C atom); however, this was only observed for very high-resolution maps (for example 1.5 Å and higher). Therefore, we did not consider normalizing to different atom types here because the majority of maps in the EMDB are at lower resolutions (Supplementary Fig. S6).

In the future, correlating *Q*-scores to local resolution and developing a percentile statistical model based on local resolution may also be interesting and useful. Comparing local *Q*-scores with local resolution is likely to be very useful as well, for example to decide whether a side chain is fitted properly (high local resolution, high *Q*-score), not fitted properly (high local resolution, low *Q*-score), overfitted (low local resolution, high *Q*-score) or if the side chain is simply not resolved in the map (low local resolution, low *Q*-score).

In the 5–10 Å resolution range, we saw that *Q*-scores decline more slowly as a function of resolution (Figs. 1 ▸ and 2 ▸). Thus, the *Q*-score is less useful in this range as it is not as sensitive to the resolution of the map. However, *Q*-scores can still be consulted for such cases to indicate potential issues. For example, a *Q*-score close to 0 can suggest that the model is not properly fitted to the map, as was seen in the example in Fig. 2 ▸(*d*). We also saw an example where the *Q*-score for a 7 Å resolution map was much higher than the commonly observed value (Fig. 2 ▸*e*). Visual inspection revealed that the map contained areas of higher resolution, so the reported resolution was not fully representative of the entire map. Thus, the current formulation of *Q*-scores may, for the time being, also be useful in this resolution range as a means of identifying such inconsistencies.

In previous work, we also noted the relation between *Q*-scores and atomic *B* factors (Pintilie & Chiu, 2021 ▸), and here we further explored and showed examples of how *Q*-scores can be converted to *B* factors at resolutions between 1 and 4 Å. We showed that when these *B* factors are used to generate a model map, the model map is more similar to the experimentally obtained 3DEM map than when not using atomic *B* factors (or setting atomic *B* factors to 0). *B* factors calculated from *Q*-scores may be inaccurate if the model is not optimally fitted to the map, or due to other factors such as radiation damage and net charge. Proper fit can be checked visually, while the other factors may be further investigated and adjusted for in future work by considering, for example, atom and residue type. With this caveat in mind, estimated *B* factors can be very useful annotations for 3DEM atomic coordinates archived in the PDB. We noted that the atomic *B* factors discussed here are different from two other *B* factors often mentioned in 3DEM: *B* factors for sharpening a 3DEM map (Terwilliger *et al.*, 2018 ▸) and the Rosenthal–Henderson *B* factors to estimate the number of particles needed for a certain resolution as constrained by instrumental and sample conditions (Rosenthal & Henderson, 2003 ▸).

To assess 3DEM entries in the EMDB (wwPDB Consortium, 2024 ▸), we have also described two percentile-based metrics here: *Q*_relative_all and *Q*_relative_resolution. The *Q*_relative_all metric represents the overall quality of the map and model, comparing their *Q*-score with the entire EMDB archive. The higher the *Q*_relative_all metric is, the higher the quality of the map and model. On the other hand, *Q*_relative_resolution compares the *Q*-score of a map and model with the *Q*-scores of other entries of similar resolution. For this score, the closer it is to 50%, the more it is ‘as commonly observed’ for other entries in the EMDB of similar resolution. *Q*_relative_resolution scores that are much higher (for example above 95%) or much lower (for example less than 5%) could indicate inconsistencies such as poorly fitted models, oversharpened maps, overfitted models or reported resolutions that may not fully reflect the entire map. *Q*-scores are already reported on EMDB entry web pages and in the wwPDB validation report. The *Q*_relative_all and *Q*_relative_resolution scores described in this study are also reported on EMDB entry web pages and are for eventual display in wwPDB Validation Reports of 3DEM structures archived in the PDB.

Finally, we note that *Q*-scores do not evaluate the stereochemical quality of an atomic coordinate model, such as proper bond lengths, bond angles, dihedral angles, chiral centers *etc*. These attributes are currently evaluated using other methods such as *MolProbity* (Williams *et al.*, 2018 ▸) and reported within EMDB validation reports. Within the wwPDB OneDep system, the same methods are used to assess structures determined using 3DEM, MX and nuclear magnetic resonance spectroscopy to support deposition and rigorous validation (Gore *et al.*, 2017 ▸; Feng *et al.*, 2021 ▸; Young *et al.*, 2017 ▸). We hope that *Q*-scores will continue to serve as a complementary and necessary metric alongside such other metrics to reflect 3DEM map–model fit and map quality.

## Supplementary Material

Supplementary Figures, Q-score distributions with outlier removal, Q-score distribution around a polynomial regression curve and Q-score distribution using a rolling window. DOI: 10.1107/S2059798325005923/ic5125sup1.pdf

## Figures and Tables

**Table 1:** Numbers of entries (#Entries; with minimum, mean and maximum) for different window sizes across resolutions of 1–10 Å

Window size *w* (A)	0.1	0.2	0.3	0.4	0.5	0.6	0.7	0.8	0.9	1	1.2	1.5
Minimum #Entries	12	26	39	43	55	73	100	159	237	299	360	453
Mean #Entries	2056	3744	5109	6761	8288	9584	10740	11791	12751	13639	14992	16364
Maximum #Entries	3651	6214	7952	10282	12358	13905	15044	15922	16681	17238	18057	18698

**Table 2:** Entries from Figs. 2 ▸ and 3 ▸ show corresponding *Q*-score, *Q*_relative_all and *Q*_relative_resolution *Q*_relative_resolution is calculated using a resolution-window size of 0.5 Å.

EMDB ID	Resolution (Å)	*Q*-score	*Q*_relative_all	*Q*_relative_resolution
EMD-7770	1.9	0.70	99%	73%
EMD-21432	2.4	0.075	3.9%	0.39%
EMD-22136	2.9	0.51	71%	51%
EMD-31385	3.1	0.43	47%	26%
EMD-30321	3.5	0.66	97%	99%
EMD-0449	5.7	0.001	0.78%	0.94%
EMD-23206	7.0	0.36	31%	100%

## Data Availability

Files with *Q*-scores and reported resolution for the plots generated here can be found in the Github repository at https://github.com/gregdp/mapq/tree/master/data. *Q*-scores and reported resolutions for map–model pairs can also be accessed at https://www.ebi.ac.uk/emdb/api/search/resolution:[*%20TO%20*]%20AND%C2%A0average_qscore_value:[*%20TO%20*]?rows=100000&wt=csv&download=false&fl=emdb_id,structure_determination_method,resolution,average_qscore_value.

## Appendix A

### A1. Methods

#### A1.1. *Q*-score calculations

*Q*-scores were calculated using the *Q*-score plugin for *UCSF Chimera* v1.9.7. The only parameter that can be varied, σ, which corresponds to the width of the reference Gaussian, was set to 0.4. Polynomial regression of *Q*-scores and reported resolution was performed in *Microsoft Excel*. Cross-correlation and cross-correlation about the mean were performed using the function overlap_and_correlation in the *FitMap* module in *UCSF Chimera* (Pettersen *et al.*, 2004 ▸).

The *Q*-score method and code is available as a plugin for *UCSF Chimera* at the following GitHub repository: https://github.com/gregdp/mapq. *Q*-scores are calculated for EMDB and wwPDB records using this implementation. The percentile scores are reported with the latest version, v2.9.7. Instructions for installation, updates and use can be found on the GitHub page. A plugin for *UCSF ChimeraX* is also available, with more limited functionality at the current time, and a slightly different calculation methodology yielding very similar per-atom *Q*-scores (https://github.com/tristanic/chimerax-qscore). The *ChimeraX* plugin can be installed from the Tools menu → More Tools → Model validation → QScore.

#### A1.2. Residual plots

*R* (https://www.r-project.org/) was used to further analyze the goodness of fit of the polynomial regression calculations and their appropriateness. Residual standard error was generated for each regression calculation, as were the residue plot and *QQ*-plot of the residuals. Examples of residual plots for average *Q*-scores of all maps and models, and maps and models with CC ≥ 0.7, are illustrated in Supplementary Fig. S1. The residual plots and *QQ*-plots were used to verify that the polynomial regression calculations are appropriate statistical models that both satisfy the assumptions and sufficiently describe the data fitting.

#### A1.3. Cross-validation

We also calculated cross-validation results, where the 10 000 map–model entries were randomly split into 90% training data and 10% testing data. Polynomial regression was performed on the training data and then verified against the testing data. The *R*^2^ of the regression model against the testing data was 0.7, consistent with the *R*^2^ of regression on the full data set and that of the training model itself. The root-mean-squared error (RMSE) of the testing data on the training model is 0.07 and the mean absolute error (MAE) is 0.05. Therefore, on average, the training model predicts *Q*-scores within ±(0.05–0.07) of the observed value for the testing data, which is low and indicates a reliable regression model.

#### A1.4. CC and CC-mean scores

Two formulations of the real-space cross-correlation scores were used: cross-correlation (CC) and cross-correlation about the mean (CC-mean).

The cross-correlation (CC) score is defined as

In equation (8), 〈〉 denotes the inner product of two vectors. The vector **m** contains values taken from the grid points in the model map. A model map is generated from atom coordinates using the *Chimera*molmap function, which takes two parameters: resolution and grid spacing. Here, for resolution we used the reported resolution of the 3DEM map associated with the model, and for the grid spacing the default, resolution/3. The molmap function simply places a Gaussian at each atom position, with height proportional to the element number of the atom and sigma proportional to the resolution, *d*: σ = *d*/(π*2^1/2^). The values in **m** are taken from the model map, but only at grid points where the values are above a threshold; here, we used 0.01. The vector **c** contains values interpolated in the 3DEM map at spatial positions corresponding to the grid points of **m**.

Using the same vectors **m** and **c**, the cross-correlation about the mean (CC-mean) is calculated as

In equation (9), **m** and **c** refer to the same vectors as described above. When they have a bar above them, they are vectors with the same size but for which each entry is the average value of the respective vector.

#### A1.5. Estimation of *B* factors with *Q*-scores

*B* factors were generated from *Q* scores using equation (5). A range of scaling factors, *f*, were applied, ranging from 0 to 300, in steps of 20. For each scaling factor, a model was generated with *B* factors according to the scaling equation (5) and then converted to a model map using *phenix.fmodel* and *phenix.mtz*2*map*. The commands and parameters are as follows: command 1, phenix.fmodel high_resolution=[map resolution] scattering_table=electron generate_fake_p1_symmetry=True [model file path]; command 2, phenix.mtz2map high_resolution=[map resolution] include_fmodel=true scattering_table=electron [model file path] [mtz file generated by command 1].

We set the [map resolution] parameter to be the same as the reported resolution of the 3DEM map with which the model map is being compared. The resulting model maps were then opened with *Chimera*. All points with values above 0.01 were considered, and the values at these points stored in a vector **m**. Values at corresponding positions were interpolated from the 3DEM map and stored in another vector **c**. The cross-correlation about the mean (CC-mean) between the vectors **m** and **c** was then calculated using the chimera.overlap_and_correlation function as per equation (9).

#### A1.6. Correlation between *Q*_relative_resolution and resolution

The correlation coefficient between *Q*_relative_resolution and the reported resolution is calculated as follows:

In equation (10), *Q*_relative_resolution_ is a vector with *Q*_relative_resolution values, while *R* is a vector with the corresponding reported resolution values. Here, *Q*_relative_resolution_ and *R* correspond to the mean values of *Q*_relative_resolution and resolution, respectively. The correlation coefficient *r* is calculated separately for two groups based on resolution: one group includes observations with a resolution smaller than 5 Å and the other comprises those with a resolution of 5 Å or greater. This approach allows the assessment of correlation across different resolution ranges.
