# Supplementary material for: Q-score as a reliability measure for protein, nucleic acid and small-molecule atomic coordinate models derived from 3DEM maps
Source: Acta Crystallogr D Struct Biol. 2025 Jul 14;81(Pt 8):410–22. doi: 10.1107/S2059798325005923 (PMC12315582; doi:10.1107/S2059798325005923)
Supplement: Supplementary file 1 [file d-81-00410-sup1.pdf]

STRUCTURAL BIOLOGY

Volume 81 (2025)

Supporting information for article:

Q-score as a reliability measure for protein, nucleic acid and small-molecule atomic coordinate models derived from 3DEM maps

Grigore Pintilie, Chenghua Shao, Zhe Wang, Brian P. Hudson, Justin W. Flatt, Michael F. Schmid, Kyle L. Morris, Stephen K. Burley and Wah Chiu

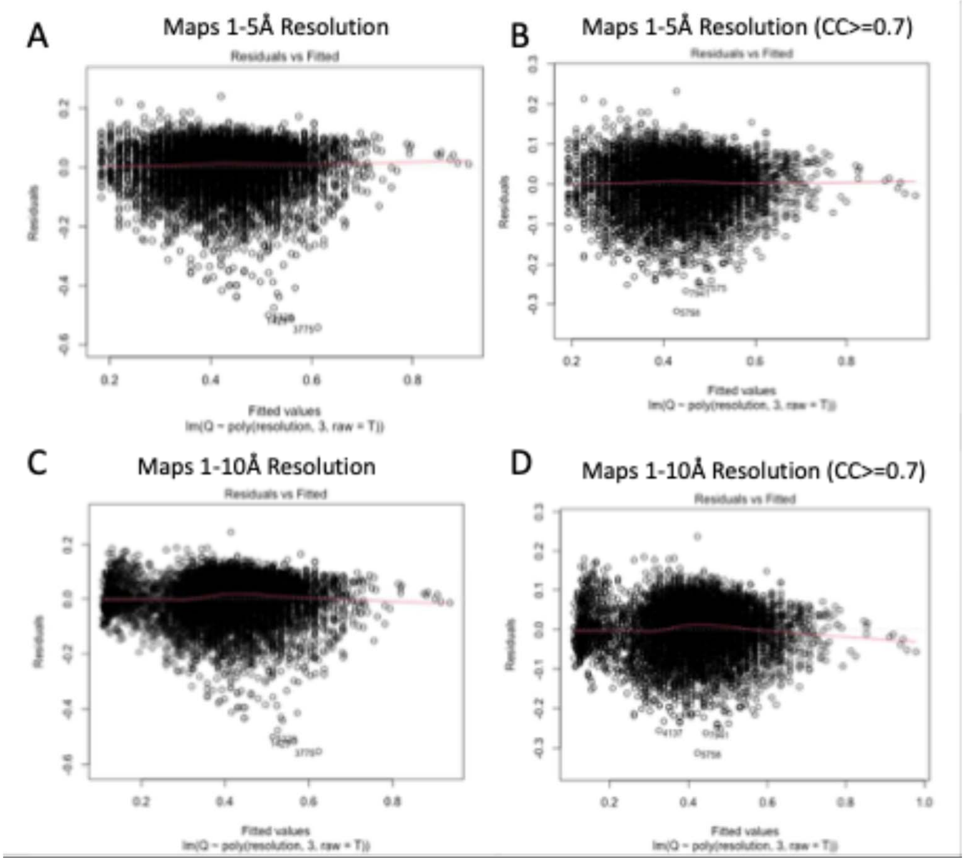

Figure S1 Residual plots for the regression calculation in Figure 1 of average Q-score vs. Reported Resolution, for (A) map-model pairs at 1-5Å resolution and (B) map-model pairs at 1-5Å resolution with map-model CC above 0.7. Residuals are well-distributed indicating the regression calculation is appropriate for this resolution range. Residual plots for the same regression calculations, but with maps in the resolution range 1-10Å are shown in (C) for all maps, and in (D) for maps with map-model pair CC above 0.7. An informative regression calculation should produce an unbiased and homoscedastic residual plot without any obvious pattern. Plot (B) indicates the best regression calculation among the four, and the other three plots demonstrates minor deviation from a perfect regression calculation, with plot (A) impacted by the outliers, and plots (C) and (D) impacted by the relatively different distribution for the resolution ranges of 1-5Å and 5-10Å. The residual standard errors are 0.075 for all maps with resolution 1-5 Å (A), 0.061 for maps with resolution 1-5Å and map-model pair CC >= 0.7 (B), 0.074 for all maps with resolution 1-10 Å (C), and 0.062 for maps with resolution 1-10Å and map-model pair CC>=0.7 (D). Both X and Y axes are scaled.

S1. Q-score distributions with outlier removal

While many map-model Q-scores appear close to the polynomial fit curve in Figure 1A, there are also some that are quite far from it. We tested whether removing some maps and models would change the correlation between Q-scores and reported resolution, aiming to remove maps and models that do not match well due to reasons such as inaccurate modeling. We applied two model-map metrics which measure the similarity between the 3DEM map and a model-computed map: cross-correlation about the mean (CC-mean) and cross-correlation (CC); these are further described in Methods. These two metrics are used because they can potentially identify which models are not fitted properly to the map(Pintilie and Chiu 2012). Higher scores mean that the model matches the map better, and that it fits well. Lower scores mean the model does not match the map as well and could potentially be incorrectly fitted.

Figures S1A-C show plots of Q-score vs. reported resolution before and after removing maps-model pairs with CC-mean<0.5 and CC<0.7. Different thresholds were used for CC-mean (threshold = 0.5) and CC (threshold = 0.7) in order to remove a similar number of entries from the datasets (~1,000 entries). The chosen threshold values are different since CC scores tend to be higher than CC-mean scores. This is because when subtracting the mean for the CC-mean score, map values are lower, some becoming negative. The remaining map-model pairs have Q-scores much closer to the fitted polynomial regression curve, as plotted in Figures S1B and S1C. Figure S2E and S2F show Q-scores vs. resolution for the removed map-model pairs. When map-model pairs are removed because of CC-mean<0.5, the remaining map-model pairs have Q-scores mostly below the regression curve, while for the map-model pairs removed based on CC<0.7, many are close to the curve as well. The regression plots in Supplementary Figures S1H and S1I show that CC-mean scores are weakly correlated to Q-scores (R^2=0.3264), while CC scores appear to have no correlation (R^2=0.0071).

The plot for a small dataset with 386 map-model pairs from the EMDB/PDB reported previously(Burley et al. 2022) is shown in Supplementary Figure S2D. The regression curves for these 386 map-model pairs, for the data set of ~10k map-model pairs, and for the data sets after removal of low CC and CC-mean scores are all plotted together for comparison in Supplementary Figure S2G, showing they are all very similar. Thus, the relationship between Q-score and reported resolution appears to be robust for different but representative data sets, and after removing some map-model pairs with low similarity (using CC-mean and CC scores).

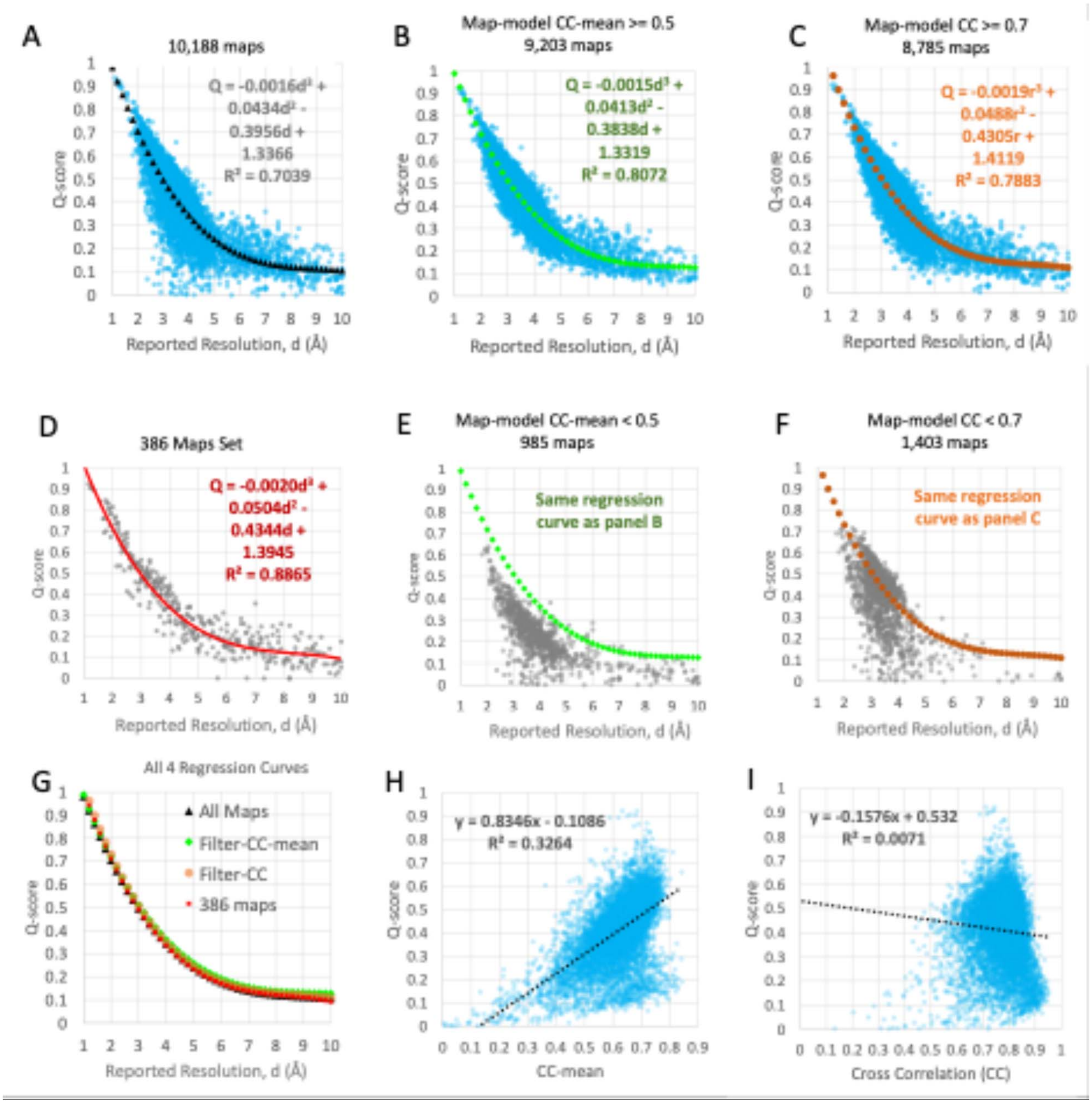

Figure S2 Regression analysis of Q-scores vs. reported resolution for 4 data sets (A-D). (A) 10,189 map-model pairs used in this analysis; note that this is the same dataset shown in Figure 1A. (B) Same data set as (A), but only maps and models with CC-mean >= 0.5. (C) Same as (A) but only maps and models with CC >= 0.7. (D) 386 maps-model pairs previously reported. (E) Map-model pairs with CC-mean < 0.5, with regression curve from panel B. (F) Map-model pairs with CC < 0.7 with regression

curve from panel C. (G) All 4 regression curves for (A-C) and (D) plotted together. (H,I) Q-score vs. CC-mean and CC, with linear regression lines.

S2. Q-score Distribution Around Polynomial Regression Curve

We further characterize the distributions of Q-scores above and below the 3rd degree polynomial regression curve, with the aim of identifying outliers which are far from this regression line. We looked at how many maps and models have Q-scores within a small window of the curve when it is moved by an offset along the vertical Q-score axis. This offset acts to move the curve up and down. At each offset, we count the number of map-model pair data points close to the curve at the offset position, within a small window size, e.g. w=0.01, as illustrated in Supplementary Figure S3A. Supplementary Figure S3B plots the number of maps and models for offsets in the range [-0.3, 0.3]. This range of offset was used because beyond +/-0.3, there are very few Q-scores around the offset curve. The plot in Supplementary Figure S3B shows that the 0-offset regression curve, which represents the mean or average Q-score, is slightly below the median which is found at offset=0.012, and the peak which is found at offset=0.024. The median represents the offset at which half of the maps have Q-scores below the curve and the other half have higher Q-scores, while the peak represents the offset at which the highest number of maps have corresponding Q-score.

Supplementary Figure S3B shows that the Q-score distribution around the polynomial regression curve is close to a normal distribution, although it is left-skewed, i.e. it decreases more slowly on the left side. The top and bottom offsets on either side of the peak which enclose 95% of the data are marked in Supplementary Figure S3C. The regression curve is plotted with corresponding offsets for the mean, median, and peak in Supplementary Figure S3D, which are very similar, and also at the offset values enclosing 95% of the data.

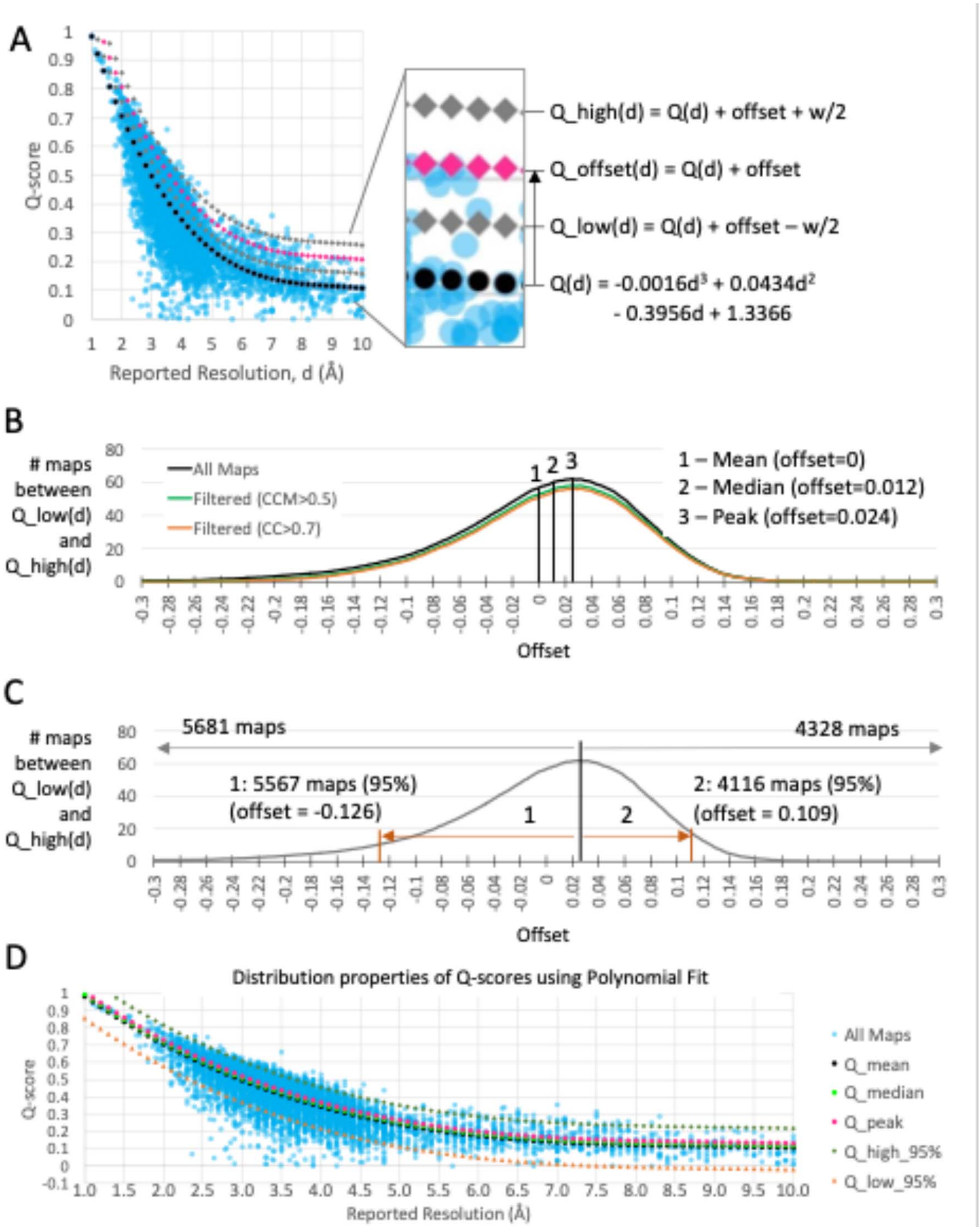

Figure S3 Distribution of Q-scores based on the 3rd degree polynomial regression curve. (A) The regression curve for Q-scores vs. reported resolution, Q(d), is plotted with a dotted black line. The same curve is also plotted with an offset, and upper and lower bounds using window size w. (B) The number of maps with Q-scores within a window of size w around the offset is plotted (w=0.01), vs. the offset ranging between [-0.3,0.3]. Q-scores of the mean, median, and peak are marked on the plot. (C) Two

offsets are marked on the curve which enclose 95% of the maps around the peak Q-score value. Note that plots in B and C include all data points. (D) The regression curves representing mean, median, and peak are plotted. Curves enclosing top and bottom 95% of the maps above and below the peak are also plotted.

S3. Q-score Distribution Using Rolling Window

We further investigated Q-score distribution using a rolling window over the resolution range 1.0 to 10 Å. This allows us to examine Q-score distribution at each resolution without the need of a regression curve fitted to all the data points. At each resolution d, maps and models with resolution d-w/2 to d+w/2 are considered, as illustrated for resolution d=2.5 Å in Supplementary Figure S4A. The number of maps and models with resolution within this range vs. Q-score is plotted in Supplementary Figure S4B. The curve is close to a normal distribution, but also decreasing more slowly on the left (towards lower Q-scores). The mean, median, and peak are calculated for this distribution and shown on the plot in Supplementary Figure S4B. The top and bottom Q-scores within which 95% of the maps and models in this resolution range are included are also calculated, and marked on the curve in Supplementary Figure S4B. This procedure was repeated for resolutions in the range 1-10 Å; all the peak and 95% thresholds at each resolution are plotted in Supplementary Figure S4C. They are similar to the smoother polynomial regression curves with corresponding offsets, as shown in Supplementary Figure S4D. The values obtained with the rolling window are however discontinuous, especially in the resolution range 1.0 to 2.0 Å, where there are fewer maps and models.

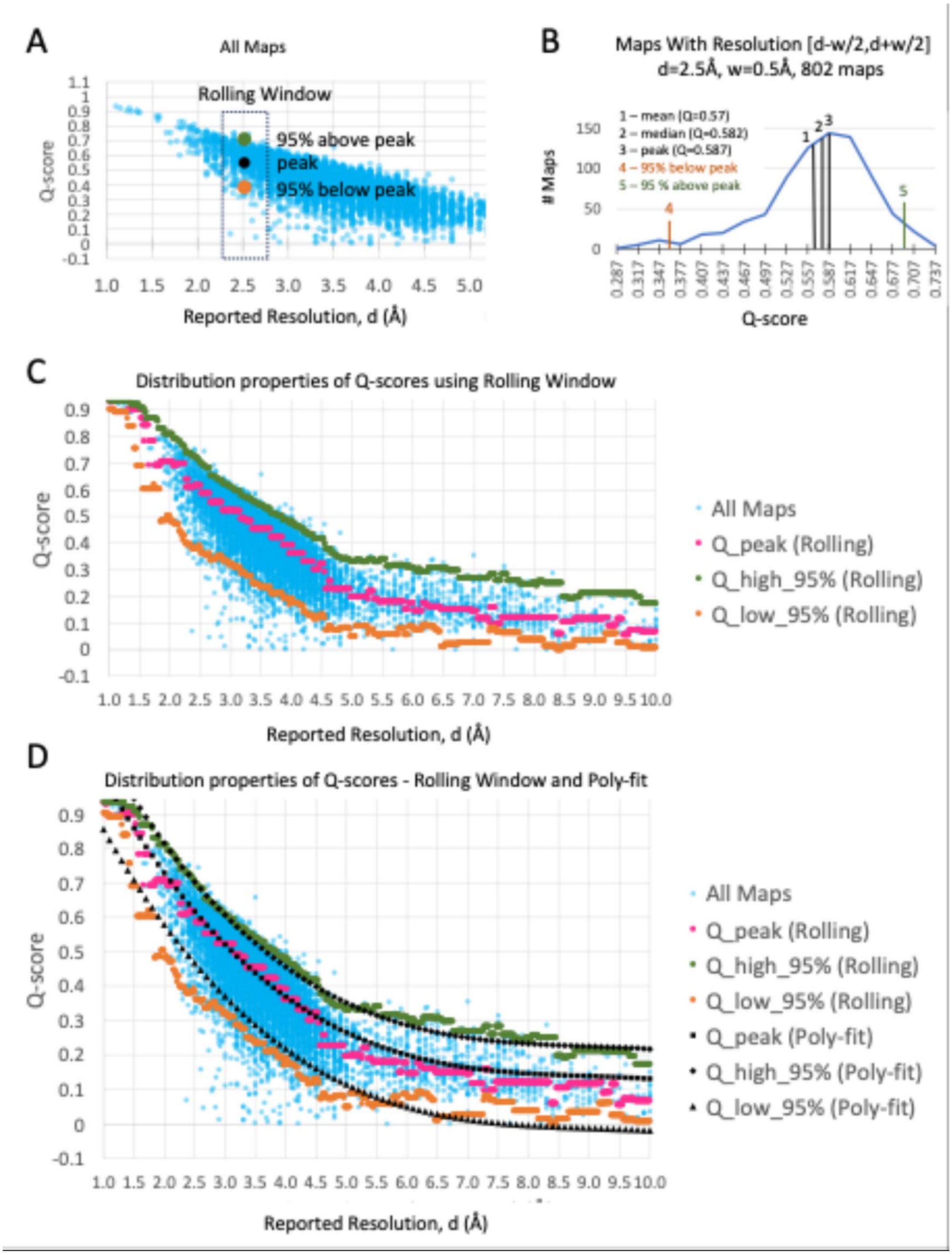

Figure S4 Calculation of mean, median, peak, and percentiles using rolling window approach. (A) Plot of Q-score vs. resolution for a subset of the ~10k maps and models with resolution < 5.0Å. A window positioned at reported resolution d=2.5Å is shown, with peak and 95% percentile points. (B) Distribution curve for the 802 maps within a window of size w=0.5Å, with mean, median, peak, and 95% percentiles

marked. (C) Plot of all ~10k maps and models vs. resolution, showing the mean, median, peak, and 95% percentile points obtained at each resolution using rolling windows. (D) Comparison of rolling window (Rolling) results with the polynomial regression curves (Poly-fit).

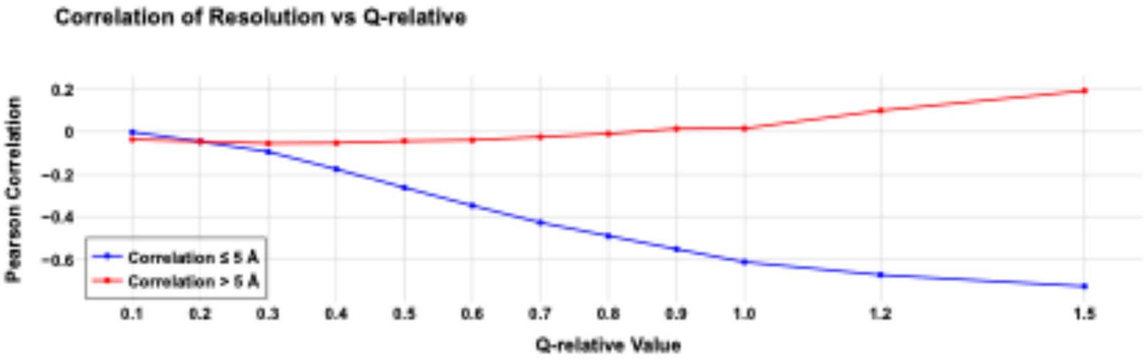

Figure S5 Plot of Pearson correlation between Q-relative-resolution and reported map resolution vs. resolution window sizes. The blue curve is for maps in EMDB with reported resolution ≤ 5 Å, while the red curve is for maps in EMB with reported resolution > 5 Å.

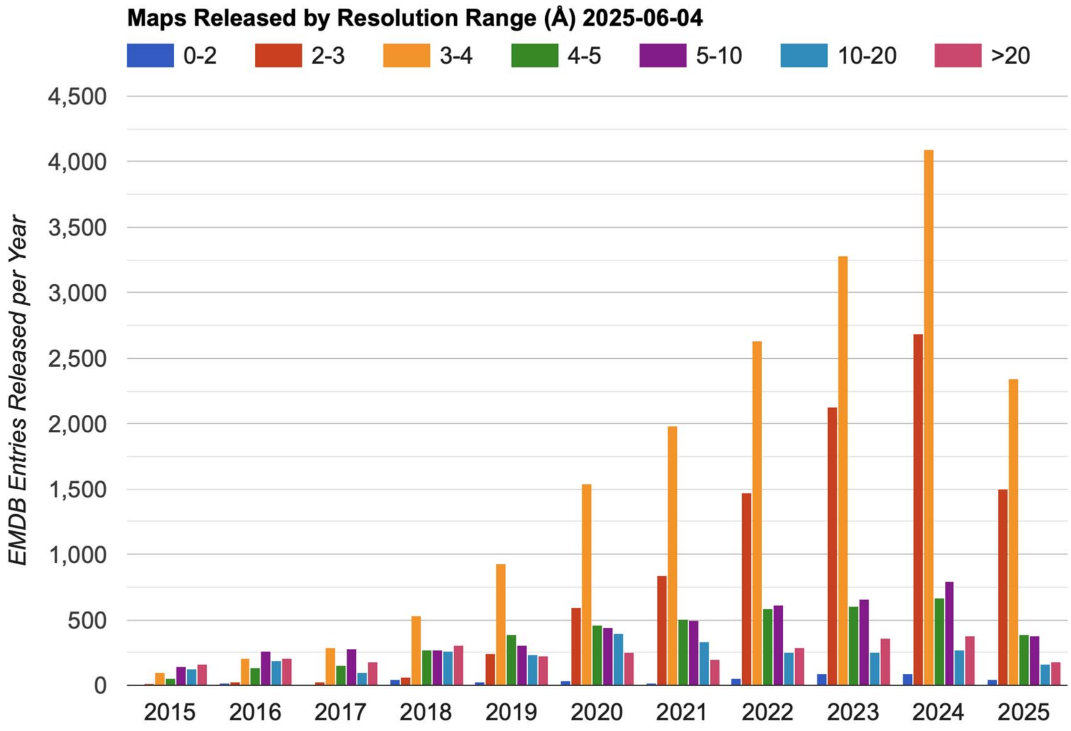

Figure S6 *Distribution of maps in the EMDB with different resolutions. Source:* https://www.emdataresource.org/statistics.html*, June 2025.*
